# COVID-19 e Estado de Hipercoagulabilidade: Uma Nova Perspectiva Terapêutica

**DOI:** 10.36660/abc.20200308

**Published:** 2020-05-22

**Authors:** Jorge Henrique Paiter Nascimento, Bruno Ferraz de Oliveira Gomes, Plínio Resende do Carmo, João Luiz Fernandes Petriz, Stephanie Itala Rizk, Isabela Bispo Santos da Silva Costa, Marcus Vinicius Guimarães Lacerda, Fernando Bacal, Ludhmila Abrahão Hajjar, Gláucia Maria Moraes de Oliveira

**Affiliations:** 1 Universidade Federal do Rio de Janeiro Rio de JaneiroRJ Brasil Universidade Federal do Rio de Janeiro – Cardiologia,Rio de Janeiro, RJ - Brasil; 2 Rede D’Or São Luiz Rio de JaneiroRJ Brasil Rede D’Or São Luiz – CardiologiaRio de Janeiro, RJ - Brasil; 3 Hospital Barra D’Or Rio de JaneiroRJ Brasil Hospital Barra D’Or – Cardiologia,Rio de Janeiro, RJ - Brasil; 4 Universidade de São Paulo Instituto do Câncer do Estado de São Paulo São PauloSP Brasil Universidade de São Paulo Instituto do Câncer do Estado de São Paulo,São Paulo, SP - Brasil; 5 Universidade de São Paulo Instituto do Coração São PauloSP Brasil Universidade de São Paulo Instituto do Coração,São Paulo, SP - Brasil; 6 Hospital Sírio-Libanês Instituto Sírio Libanês de Ensino e Pesquisa São PauloSP Brasil Hospital Sírio-Libanês - Instituto Sírio Libanês de Ensino e Pesquisa,São Paulo, SP - Brasil; 7 Fundação de Medicina Tropical Dr Heitor Vieira Dourado ManausAM Brasil Fundação de Medicina Tropical Dr Heitor Vieira Dourado,Manaus, AM - Brasil; 8 Universidade de São Paulo Faculdade de Medicina Hospital das Clínicas Instituto do Coração São PauloSP Brasil Universidade de São Paulo Faculdade de Medicina Hospital das Clínicas Instituto do Coração,São Paulo, SP - Brasil; 9 Intituto de Coração São PauloSP Brasil Intituto de Coração – Cardiopneumologia,São Paulo, SP – Brasil

**Keywords:** COVID-2019, Betacoronavirus, Doença Catastrófica, Pneumonia Viral, Pandemias, Infecções por Coronavirus, Complicações Cardiovasculares, Trombofilia, Anticoagulantes/uso terapêutico

O novo coronavírus, designado como vírus da síndrome respiratória aguda grave (SARS-CoV-2, sigla em inglês), é o responsável pelo surto da pneumonia viral que foi identificada pela primeira vez na cidade chinesa de Wuhan ao final de 2019 e que rapidamente se espalhou acometendo 184 países. A experiência adquirida nos últimos meses descreve diferentes apresentações clínicas com gravidade variável, desde infecção assintomática até óbito por disfunção orgânica múltipla. Recentemente, a Organização Mundial de Saúde (OMS) definiu o complexo processo de adoecimento por esse vírus como doença causada pelo coronavírus 2019 (COVID-19, sigla em inglês).

A COVID-19, cuja notificação é crescente em diferentes países, atualmente afeta mais de um milhão de pessoas, segundo a OMS, que a caracterizou como pandemia.^1^ No Brasil, até o dia 29 de abril de 2020, foram confirmados 73.235 casos da infecção e 5.083 óbitos, com taxa de letalidade de 6,9%.^2^ A hospitalização é necessária em até 20% dos pacientes infectados por SARS-CoV-2, e 5% a 10% deles terão indicação de internação em terapia intensiva por necessidade de suporte hemodinâmico e/ou ventilatório.^3-7^ A taxa de mortalidade varia de 0,8% a 12% de acordo com o país, possivelmente como consequência de múltiplos fatores, dos quais se destaca a estrutura do sistema de saúde.^8-11^Os pacientes que desenvolvem as formas moderada e grave da doença apresentam manifestações predominantemente decorrentes de acometimento do sistema respiratório, com quadro clínico variando de pneumonia leve a síndrome do desconforto respiratório agudo (SDRA).^7,11-13^

As complicações, em geral, ocorrem entre o 7º e o 12º dia da doença.^3,14^ A SDRA é a manifestação clínica mais grave e caracteriza-se por hipoxemia, infiltrado pulmonar bilateral e fenótipos variáveis de apresentação, como o perfil de complacência pulmonar normal e baixo potencial de recrutamento pulmonar até o perfil de baixa complacência pulmonar e alto potencial de recrutamento pulmonar. De 20% a 30% dos pacientes terão complicações cardiovasculares, como isquemia miocárdica, síndrome coronária aguda, miocardite, arritmias, insuficiência cardíaca e choque. Insuficiência renal ocorre em aproximadamente 30-50% dos pacientes críticos infectados pelo SARS-CoV-2 e 30% deles necessitarão de terapia de substituição renal.^14-17^

O SARS-CoV-2 entra nas células via receptor da enzima conversora de angiotensina 2 (ECA2), que está presente nos alvéolos. A forma grave da infecção é caracterizada por uma resposta inflamatória imunológica intensa, evidenciada pela presença de neutrófilos, linfócitos, monócitos e macrófagos.^18^ Necrópsias minimamente invasivas revelam dano alveolar difuso, formação de membrana hialina e infiltrado inflamatório intersticial mononuclear, com trombose em microcirculação.^17^ Foram relatados nesses pacientes níveis elevados de citocinas pró-inflamatórias (interleucinas 1 e 6, fator de necrose tumoral e interferon-g) no sangue, uma condição denominada “tempestade de citocinas”.

Trombose e danos a órgãos extrapulmonares também são observados sem a presença comprovada do vírus nos locais, postulando-se que a infecção pelo SARS-CoV-2 envolva intensa resposta inflamatória, com estado de hipercoagulabilidade e isquemia, agravados por hipoxemia.^17,19,20^ No Brasil, achados preliminares de autópsias minimamente invasivas realizadas por pesquisadores da Faculdade de Medicina de São Paulo mostraram resultados semelhantes aos encontrados na China.^21^

O dímero-D, um produto da degradação da fibrina, quando elevado, tem sido associado a maior taxa de mortalidade.^22^ A opinião de especialistas, baseada em experiência clínica e análise de poucos estudos descritivos, destaca o papel do estado de hipercoagulabilidade na fisiopatologia da COVID-19, uma vez que o nível de dímero-D aumenta progressivamente com a exacerbação da infecção. A fase da doença em que ocorre o desenvolvimento de SDRA e a piora do padrão radiológico é marcada pela elevação expressiva de dímero-D, observando-se nos casos mais graves injúria miocárdica e coagulação intravascular disseminada (CIVD).^23,24^

A resposta inflamatória sistêmica em pacientes com infecção pode resultar em lesão endotelial com consequente aumento na geração de trombina e redução da fibrinólise endógena.^25,26^ Esse estado pró-trombótico é denominado coagulopatia induzida pela sepse (SIC, sigla em inglês) e precede a CIVD.^27,28^ Os diversos mecanismos envolvidos na SIC agem simultaneamente, culminando em um estado pró-hemostático. Aparentemente, os fatores mais importantes que medeiam esse distúrbio do sistema de coagulação durante a sepse são as citocinas inflamatórias.

**Figura 1:**
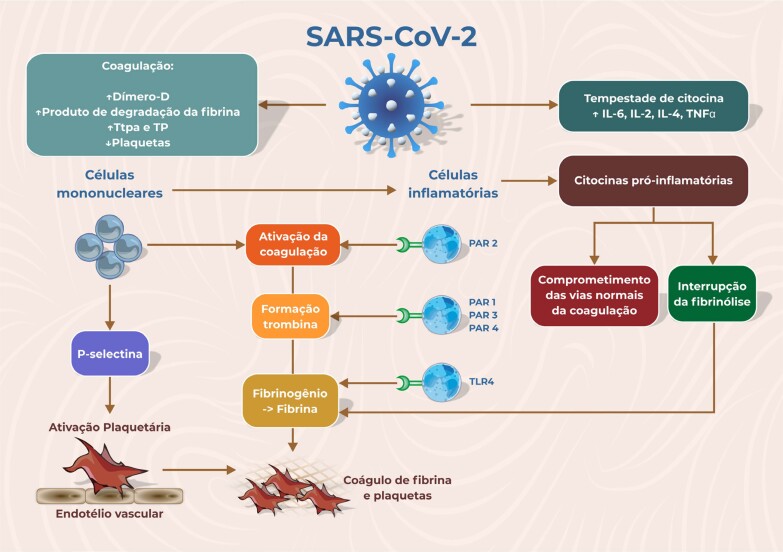
– *O novo coronavírus, SARS-CoV-2, ativa o processo inflamatório e trombótico, e a infecção por ele causada está relacionada a aumento de citocinas inflamatórias (tempestade de citocinas) e distúrbios da coagulação, com predisposição a formação de trombos. As células mononucleares interagem com as plaquetas ativadas e a cascata da coagulação, que ativam as células inflamatórias por meio da ligação da trombina e do fator tecidual com receptores específicos ativados por protease e da ligação da fibrina ao receptor Toll-like 4. A ativação das células inflamatórias resulta na liberação de citocinas pró-inflamatórias, que levam a comprometimento das vias normais da coagulação e interrupção da fibrinólise. PAR: receptor ativado por protease; TLR4: receptor Toll-like 4; Ttpa: tempo de tromboplastina parcial ativada; TP: tempo de protrombina; IL: interleucina; TNFα; fator de necrose tumoral-α. Figura adaptada de: Levi M, van der Poll T.^25^*

Evidências demonstram uma interação cruzada entre inflamação e coagulação, com a inflamação induzindo a ativação da coagulação e a coagulação acentuando a atividade inflamatória (Figura 1).^29-32^ As plaquetas têm um papel central no desenvolvimento das anormalidades da coagulação na sepse e podem ser ativadas diretamente por mediadores pró-inflamatórios, como o fator ativador de plaquetas, bem como por meio da trombina gerada. A ativação de plaquetas também pode estimular a formação de fibrina por mecanismo alternativo. A expressão de P-selectina na membrana plaquetária não apenas medeia a adesão de plaquetas a leucócitos e células endoteliais, mas também aumenta a expressão do fator tecidual nos monócitos. Em circunstâncias normais, a ativação da coagulação é controlada por três importantes vias anticoagulantes fisiológicas: o sistema antitrombina, o sistema ativado da proteína C e o inibidor da via do fator tecidual. Na sepse, todas as três vias sofrem disfunção. Em meio a todo esse desbalanço do sistema de coagulação, a fibrinólise endógena é amplamente reduzida.

De acordo com critérios estabelecidos pela Sociedade Internacional de Trombose e Hemostasia (ISTH, sigla em inglês), é possível identificar melhores desfechos clínicos nos pacientes com SIC recebendo terapia anticoagulante.^27,28^O uso de anticoagulantes, sobretudo nos pacientes em estado crítico, não é isento de riscos e pode estar relacionado a complicações hemorrágicas graves. Portanto, a indicação dessa modalidade terapêutica deve ser personalizada, respeitando os perfis de risco trombótico e hemorrágico.

A síndrome hemofagocítica (SHF) é caracterizada por uma resposta inflamatória sistêmica desencadeada pela ativação e proliferação inapropriada dos linfócitos, que ativam macrófagos e histiócitos, que fagocitam células hematológicas. Essa síndrome é associada a grande produção de citocinas inflamatórias. O quadro clínico inicial da SHF é marcado pelos achados de síndrome de resposta inflamatória sistêmica e, na suspeição de sua evolução, podem-se observar quadro neurológico, alterações na função hepática, CIVD, hepatoesplenomegalia, pancitopenia e ferritina elevada. Esse quadro pode ser desencadeado por infecções, inclusive COVID-19, na qual alguns casos apresentam grande liberação de citocinas, principalmente interleucina 6, associada a resposta inflamatória sistêmica e CIVD. Deve-se pensar em tais condições de acordo com os achados clínicos e laboratoriais, devendo ser definida uma abordagem terapêutica precoce na tentativa de reversão do quadro.^28^

A infecção pelo SARS-CoV-2 em sua apresentação mais grave marcada por disfunção orgânica, como insuficiência respiratória aguda, atende aos critérios diagnósticos de sepse.^33^ Estudos observacionais recentes correlacionam estado de hipercoagulabilidade à forma grave de COVID-19, onde SIC e/ou CIVD parecem estar presentes na maioria dos casos fatais.^3,21-23,34^ A redução da pressão arterial de oxigênio encontrada nos pacientes críticos contribui direta e indiretamente para o desenvolvimento da síndrome isquêmica.^35^ De acordo com o exposto, é razoável alinhar a fisiopatologia pró-trombótica já descrita na sepse com aspectos intrínsecos do novo coronavírus e, portanto, analisar individualmente o potencial benefício do uso de anticoagulantes em grupos selecionados de pacientes. Um estudo retrospectivo realizado no hospital de Tongji (Wuhan, China) descreveu a ocorrência de menor taxa de mortalidade nos pacientes com COVID-19 grave que fizeram uso de anticoagulante, heparina não fracionada ou heparina de baixo peso molecular (HBPM), e apresentavam escore SIC ≥ 4 e/ou dímero-D muito elevado (> 6 vezes o limite superior da normalidade).^36^

**Figura 2:**
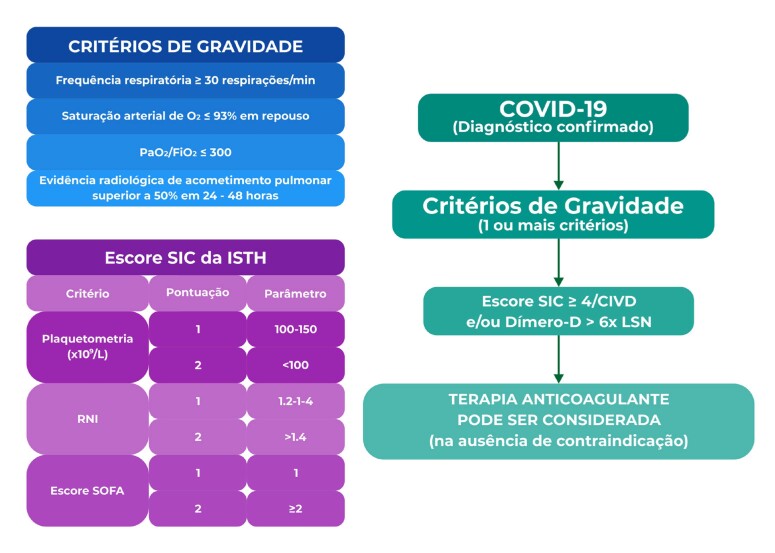
– *O diagnóstico de COVID-19 deve ser confirmado conforme as recomendações da Organização Mundial de Saúde.^37^ Pacientes com critérios de gravidade^8,38^ em associação com escore de coagulopatia induzida pela sepse ≥ 4 ou coagulação intravascular disseminada e/ou valores de dímero-D > 6 vezes o limite superior da normalidade podem se beneficiar da terapia anticoagulante. RNI: razão de normatização internacional; SIC: coagulopatia induzida pela sepse; ISTH: International Society on Thrombosis and Hemostasis; SOFA: sequential organ failure assessment; CIVD: coagulação intravascular disseminada; LSN: limite superior da normalidade.*

A terapia anticoagulante em pacientes com COVID-19 grave e indícios de SIC e/ou com dímero-D muito elevado em associação a outros biomarcadores que denotam gravidade, na ausência de contraindicação à anticoagulação, pode ser considerada uma estratégia terapêutica fundamentada no consenso de especialistas e em poucos estudos retrospectivos. Adicionalmente, essa estratégia requer a utilização de protocolos institucionais rígidos que permitam a vigilância e a rápida intervenção frente a complicações. A Figura 2 apresenta o algoritmo de avaliação da trombogênese em pacientes com COVID-19, além de uma proposta de tratamento. Não existem, todavia, dados suficientes para determinar aspectos importantes à elaboração do plano terapêutico, como a escolha da melhor droga, sua dosagem e regime de administração e a duração do tratamento.

São necessários mais estudos, sobretudo prospectivos, para melhor fundamentar a indicação da terapia anticoagulante em pacientes críticos infectados pelo novo coronavírus. O possível benefício de se reduzir o estado de hipercoagulabilidade deve ser balanceado com o risco de sangramento. É possível que a terapêutica anticoagulante seja mais benéfica quando iniciada na fase pré-trombótica do que nos quadros avançados, quando o risco de sangramento é maior. Em se optando pela anticoagulação, parece razoável o uso de HBPM como fármaco de escolha em pacientes estáveis e com depuração normal de creatinina (dose de 1 mg/kg de 12/12h, subcutânea). Em caso de choque ou depuração de creatinina abaixo de 50 ml/min/m^2^, é preferível o uso de heparina intravenosa (18 UI/kg/h), tendo como alvo um tempo de tromboplastina parcial ativada entre 1,5 e 1,8. Entretanto, não há evidências que fundamentem a ampla utilização de heparina em dose terapêutica na COVID-19.

Em conclusão, a fisiopatologia da COVID-19 envolve ativação da resposta inflamatória e indução do sistema trombótico. No momento, consenso de especialistas sugere tratamento com anticoagulante para pacientes que tenham fenótipo pró-coagulante (dímero-D elevado, prolongamento de tempo de protrombina e aumento dos níveis plasmáticos de fragmentos da fibrina). Mais estudos são necessários para confirmar o real papel da anticoagulação na prevenção de complicações da COVID-19.
