# Supplementary material for: COVID-19 e Estado de Hipercoagulabilidade: Uma Nova Perspectiva Terapêutica
Source: Arq Bras Cardiol. 2020 May 22;114(5):829–33. [Article in Portuguese] doi: 10.36660/abc.20200308 (PMC8386998; doi:10.36660/abc.20200308)
Supplement: Translation

# Translation: COVID-19 and Hypercoagulable State: A New Therapeutic Perspective

**Authors:** Jorge Henrique Paiter Nascimento, Bruno Ferraz de Oliveira Gomes, Plínio Resende do Carmo, João Luiz Fernandes Petriz, Stephanie Itala Rizk, Isabela Bispo Santos da Silva Costa, Marcus Vinicius Guimarães Lacerda, Fernando Bacal, Ludhmila Abrahão Hajjar, Gláucia Maria Moraes de Oliveira

**Affiliations:** 1 Brazil Universidade Federal do Rio de Janeiro – Cardiologia, Rio de Janeiro, RJ - Brazil; 2 Brazil Rede D’Or São Luiz – Cardiologia Rio de Janeiro, RJ - Brazil; 3 Brazil Hospital Barra D’Or – Cardiologia, Rio de Janeiro, RJ - Brazil; 4 Brazil Universidade de São Paulo Instituto do Câncer do Estado de São Paulo, São Paulo, SP - Brazil; 5 Brazil Universidade de São Paulo Instituto do Coração, São Paulo, SP - Brazil; 6 Brazil Hospital Sírio-Libanês - Instituto Sírio Libanês de Ensino e Pesquisa, São Paulo, SP - Brazil; 7 Brazil Fundação de Medicina Tropical Dr Heitor Vieira Dourado, Manaus, AM - Brazil; 8 Brazil Universidade de São Paulo Faculdade de Medicina Hospital das Clínicas Instituto do Coração, São Paulo, SP - Brazil; 9 Brasil Intituto de Coração – Cardiopneumologia, São Paulo, SP – Brasil

**Keywords:** COVID-2019, Betacoronavirus, Catastrophic Illness, Viral, Pneumonia, Pandemics, Coronavirus Infections, Complications, Cardiovasculares, Thrombophilia, Anticoagulants/therapeutic use

The novel coronavirus, named severe acute respiratory syndrome coronavirus 2 (SARS-CoV-2), is responsible for the outbreak of the viral pneumonia identified for the first time in the Chinese city of Wuhan at the end of 2019. The outbreak has expanded rapidly, affecting 184 countries. The experience acquired in past months identified different clinical presentations with varied severity, ranging from asymptomatic infection to death due to multiple organic dysfunction. The World Health Organization (WHO) has recently defined the complex process of the SARS-CoV-2 infection as novel coronavirus disease 2019 (COVID-19).

COVID-19, whose notification grows fast in different countries, currently affects more than one million people worldwide according to the WHO, which has characterized the infection as a pandemic.^1^ As of April 29, 2020, Brazil had registered 73,235 confirmed cases of COVID-19 and 5,083 deaths, with a case-fatality rate of 6,9%.^2^ Hospitalization is necessary in up to 20% of the patients infected by SARS-CoV-2, and 5% to 10% of them require admission to the intensive care unit because of the need for hemodynamic and/or ventilatory support.^3-7^ The mortality rate ranges from 0.8% to 12% depending on the country, and this difference might result from multiple factors, of which the healthcare system structure stands out.^8-11^ Patients with the moderate and severe forms of the disease had manifestations mainly of the respiratory system involvement, with clinical findings ranging from mild pneumonia to acute respiratory distress syndrome (ARDS).^7,11-13^

Complications usually occur between the 7th and 12th day of disease.^3,14^ The most severe clinical manifestation, ARDS, is characterized by hypoxemia, bilateral pulmonary infiltrate, and variable phenotypic presentations, such as ‘normal lung compliance and low potential for lung recruitment’ and ‘low lung compliance and high potential for lung recruitment’. From 20% to 30% of the patients have cardiovascular complications, such as myocardial ischemia, acute coronary syndrome, myocarditis, arrhythmias, heart failure and shock. Kidney failure occurs in 30-50% of critically ill patients infected by SARS-CoV-2, 30% of whom require renal replacement therapy.^14-17^

The SARS-CoV-2 enters the cell via the angiotensin-converting enzyme 2 (ACE2) receptor present in the alveoli. The severe form of the infection is characterized by an intense immune-inflammatory response, evidenced by the presence of neutrophils, lymphocytes, monocytes and macrophages.18 Minimally invasive autopsies have revealed diffuse alveolar damage, hyaline membrane formation and interstitial mononuclear inflammatory infiltrate, with microcirculatory thrombosis.^17^ High serum levels of pro-inflammatory cytokines (interleukins 1 and 6, tumor necrosis factor and interferon-g), known as “cytokine storm”, have been reported in those patients.

Thrombosis and damage to extrapulmonary organs have been observed without the confirmed presence of the virus in those sites, which led to the assumption that SARS-CoV-2 infection involves intense inflammatory response with a hypercoagulable state and ischemia, aggravated by hypoxemia.^17,19,20^ In Brazil, preliminary findings of minimally invasive autopsies performed at the São Paulo Medical School have shown similar results to those from China.^21^

When elevated, D-dimer, a product of fibrin degradation, has been associated with a higher mortality rate.^22^ Expert opinion, based on clinical experience and analysis of a few descriptive studies, highlights the role of the hypercoagulable state on the pathophysiology of COVID-19, supported by the progressive increase in D-dimer levels as the disease worsens. The phase in which ARDS develops and the radiographic pattern worsens is marked by the significant elevation of D-dimer. The most severe cases develop myocardial injury and disseminated intravascular coagulation (DIC).^23,24^

Systemic inflammatory response in patients with infection can result in endothelial damage, with a consequent increase in thrombin generation and a reduction in endogenous fibrinolysis.^25,26^ This prothrombotic state is called sepsis-induced coagulopathy (SIC) and precedes DIC.^27,28^ The several mechanisms involved in SIC act simultaneously towards a pro-hemostatic state. Apparently, inflammatory cytokines are the most important factors mediating that coagulation system disorder during sepsis.

Evidence has shown a bidirectional relationship between inflammation and coagulation, in which inflammation activates coagulation, and coagulation heightens inflammatory activity (Figure 1).^29-32^ Platelets play a central role in the development of coagulation abnormalities in sepsis and they can be activated directly by pro-inflammatory mediators, such as platelet activating factors, as well as by the thrombin generated. Platelet activation can also stimulate the formation of fibrin via an alternative mechanism. The expression of P-selectin in platelet membrane not only mediates the adhesion of platelets to leukocytes and endothelial cells, but also increases the tissue factor expression in monocytes. Under normal circumstances, the activation of coagulation is controlled by three important physiological anticoagulant pathways: the antithrombin system, the activated C-protein system, and the tissue-factor pathway inhibitor. In sepsis, all three pathways are dysfunctional. Amidst all this coagulation system imbalance, endogenous fibrinolysis is largely reduced.

**Figure 1:** – *The novel coronavirus, SARS-CoV-2, activates the inflammatory and thrombotic process. The disease it causes is associated with an increase in inflammatory cytokines (cytokine storm) and coagulation disorders, with predisposition to thrombus formation. Mononuclear cells interact with activated platelets and the coagulation cascade, which activate inflammatory cells by binding thrombin and tissue factor with specific protease activated receptors and by binding fibrin to Toll-like receptor 4. The activation of inflammatory cells results in the release of pro-inflammatory cytokines, leading to impairment of the natural coagulation pathways and shut down of fibrinolysis. PAR: protease-activated receptor; TLR4: Toll-like receptor 4; aPTT: activated partial thromboplastin time; PT: prothrombin time; IL: interleukin; TNFα: tumor necrosis factor-α. Figure adapted from Levi M, van der Poll T.^25^*

According to the criteria established by the International Society on Thrombosis and Hemostasis (ISTH), better clinical outcomes can be identified in patients with SIC on anticoagulant therapy.^27,28^ The use of anticoagulants, mainly in critically ill patients, is not free from risk and might be related to severe hemorrhagic complications. Thus, the indication of anticoagulants should be personalized, respecting thrombotic and hemorrhagic risk profiles.

Hemophagocytic syndrome (HPS) is characterized by a systemic inflammatory response triggered by the inappropriate activation and proliferation of lymphocytes, which activate macrophages and histiocytes, resulting in phagocytosis of hematological cells. The disease is associated with a large production of inflammatory cytokines. The initial clinical findings of HPS are marked by those of the systemic inflammatory response syndrome. As HPS develops, the following might be observed: neurological findings, liver function changes, DIC, hepatosplenomegaly, pancytopenia, and high ferritin levels. Those findings can be triggered by infections, such as COVID-19, which shows, in some cases, a large release of cytokines, mainly interleukin 6, in association with systemic inflammatory response and DIC. Such conditions should be considered based on clinical and laboratory findings, and an early therapeutic approach should be defined to reverse them.^28^

SARS-CoV-2 infection, in its most severe presentation, marked by organic dysfunction, such as acute respiratory failure, meets the diagnostic criteria for sepsis.^33^ Recent observational studies have correlated the hypercoagulable state with the severe form of COVID-19, in which SIC and/or DIC seem to be present in most fatal cases.^3,21-23,34^ The reduction in oxygen arterial pressure found in critical patients contributes directly and indirectly to the development of ischemic syndrome.^35^ In line of this, results suggest that the prothrombotic pathophysiology already described in sepsis might be related to intrinsic aspects of the novel coronavirus, and, thus, the beneficial potential of the use of anticoagulants in selected groups of patients should be analyzed individually. A retrospective study conducted in the hospital of Tongji (Wuhan, China) has reported lower mortality in patients with severe COVID-19 who had used anticoagulants, unfractionated heparin or low-molecular weight heparin (LMWH), with a SIC score of ≥ 4 and/or very high D-dimer (> 6 times the upper limit of reference range).^36^

Anticoagulant therapy in patients with severe COVID-19 and signs of SIC and/or very high D-dimer in association with other biomarkers of severity, in the absence of contraindication to anticoagulation, can be considered a therapeutic strategy for SARS-CoV-2 infection, based on expert consensus and a few retrospective studies. Moreover, that strategy requires the use of strict institutional protocols that enable surveillance and rapid intervention if complications occur. Figure 2 shows the algorithm to assess thrombogenesis in patients with COVID-19, as well as a suggestion of treatment. However, data are still insufficient to determine important aspects for the elaboration of a therapeutic plan, such as the best drug choice, its dosage and administration time schedule, as well as the duration of treatment.

**Figure 2:** – *The diagnosis of COVID-19 should be confirmed according to the World Health Organization recommendations.37 Patients with the severe form of that disease8,38 in addition to a sepsis-induced coagulopathy score ≥ 4 or disseminated intravascular coagulation and/or D-dimer levels > 6 times the upper limit of reference range might benefit from anticoagulant therapy. INR, international normalized ratio; SIC, sepsis-induced coagulopathy; ISTH, International Society on Thrombosis and Hemostasis; SOFA, sequential organ failure assessment; DIVC, disseminated intravascular coagulation; ULRR, upper limit of reference range.*

Further studies, mainly prospective, are required to better support the indication of anticoagulation for critical patients infected by the novel coronavirus. The possible benefit from attenuating the hypercoagulable state should be balanced against the risk of bleeding. Anticoagulant therapy might be more beneficial when initiated in the pre-thrombotic stage and not in advanced phases, when the risk of bleeding is higher. If deciding to use anticoagulation, LMWH should be chosen for stable patients with normal creatinine clearance (dose of 1 mg/kg, 12/12h, subcutaneous). In case of shock or creatinine clearance below 50 mL/min/m^2^, intravenous heparin (18 IU/kg/h) should be used, aiming at an activated partial thromboplastin time between 1.5 and 1.8. However, there is no evidence to support the wide use of the therapeutic dose of heparin in COVID-19.

In conclusion, the pathophysiology of COVID-19 involves activation of the inflammatory response and induction of the thrombotic system. Currently, the expert consensus suggests anticoagulant treatment for patients with the pro-coagulant phenotype (high D-dimer, prolongation of prothrombin time and increased plasma levels of fibrin fragments). Further studies are required to confirm the real role of anticoagulation to prevent COVID-19 complications.
